# 2-Hydr­oxy-6,6-dimethyl­bicyclo­[3.1.1]heptane-2-carboxylic acid

**DOI:** 10.1107/S1600536809041385

**Published:** 2009-10-17

**Authors:** Yan-Qing Gao, Shi-Bin Shang, Xu Xu, Xiao-Ping Rao, Hong-Xiao Wang

**Affiliations:** a Institute of Chemical Industry of Forest Products, Chinese Academy of Forestry, Nanjing 210042, People’s Republic of China

## Abstract

The title compound, C_10_H_16_O_3_, with a bicyclo­[3.1.1]heptane unit, was obtained by oxidation of β-pinene. The asymmetric unit contains two independent mol­ecules with similar geometry: the six-membered rings in both mol­ecules adopt envelope conformations. In the crystal, the independent mol­ecules exist as O—H⋯O hydrogen-bonded dimers. The dimers are linked into helical chains along the *b* axis by O—H⋯O hydrogen bonds.

## Related literature

For the preparation of nopinone and nopinic acid, see: Winstein & Holness (1955 ▶); Ma *et al.* (2007 ▶). For the crystal structure of sodium nopinate [sodium (1*R*,2S,5*S*)-2-hydr­oxy-6,6-dimethyl­bicyclo­[3.1.1]heptane-2-carboxyl­ate penta­hydrate], see: Ma *et al.* (2008 ▶). 
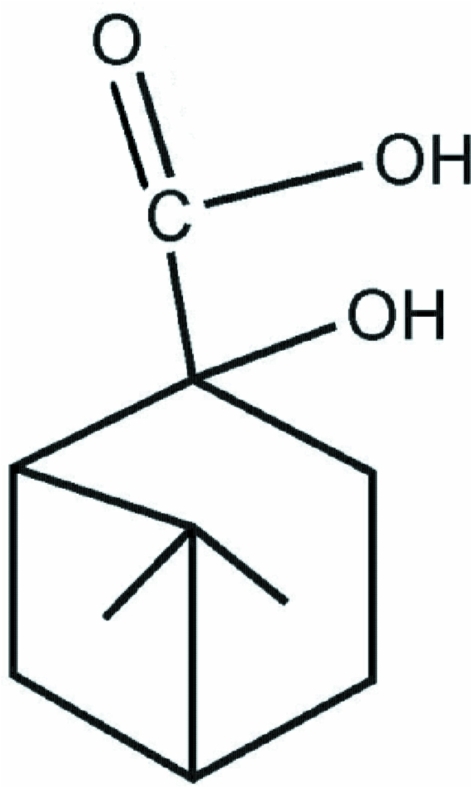


## Experimental

### 

#### Crystal data

C_10_H_16_O_3_
*M* *_r_* = 184.23
Monoclinic, 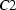

*a* = 26.796 (5) Å
*b* = 6.6560 (13) Å
*c* = 12.250 (3) Å
β = 112.23 (3)°
*V* = 2022.5 (9) Å^3^
*Z* = 8
Mo *K*α radiation
μ = 0.09 mm^−1^
*T* = 293 K
0.30 × 0.20 × 0.20 mm

#### Data collection

Enraf–Nonius CAD-4 diffractometer
Absorption correction: ψ scan (*XCAD4*; Harms & Wocadlo, 1995 ▶) *T*_min_ = 0.974, *T*_max_ = 0.983
2047 measured reflections
2002 independent reflections
1565 reflections with *I* > 2σ(*I*)
*R*_int_ = 0.018
3 standard reflections every 200 reflections intensity decay: 1%

#### Refinement

*R*[*F* ^2^ > 2σ(*F* ^2^)] = 0.056
*wR*(*F* ^2^) = 0.153
*S* = 1.00
2002 reflections
242 parameters
3 restraints
H atoms treated by a mixture of independent and constrained refinement
Δρ_max_ = 0.21 e Å^−3^
Δρ_min_ = −0.23 e Å^−3^

### 

Data collection: *CAD-4 Software* (Enraf–Nonius, 1989 ▶); cell refinement: *CAD-4 Software*; data reduction: *XCAD4* (Harms & Wocadlo, 1995 ▶); program(s) used to solve structure: *SHELXS97* (Sheldrick, 2008 ▶); program(s) used to refine structure: *SHELXL97* (Sheldrick, 2008 ▶); molecular graphics: *SHELXTL* (Sheldrick, 2008 ▶); software used to prepare material for publication: *SHELXL97*.

## Supplementary Material

Crystal structure: contains datablocks I, global. DOI: 10.1107/S1600536809041385/ci2928sup1.cif

Structure factors: contains datablocks I. DOI: 10.1107/S1600536809041385/ci2928Isup2.hkl

Additional supplementary materials:  crystallographic information; 3D view; checkCIF report

## Figures and Tables

**Table 1:** Hydrogen-bond geometry (Å, °)

*D*—H⋯*A*	*D*—H	H⋯*A*	*D*⋯*A*	*D*—H⋯*A*
O1—H1*A*⋯O2	0.81 (3)	2.38 (8)	2.837 (5)	116 (7)
O2—H2*D*⋯O6	0.82	1.80	2.621 (5)	175
O4—H4*C*⋯O1^i^	0.84 (5)	2.05 (5)	2.830 (5)	156 (5)
O5—H5*C*⋯O3	0.82	1.88	2.704 (4)	177

Symmetry code: (i) 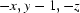.

**Fig. 1.**
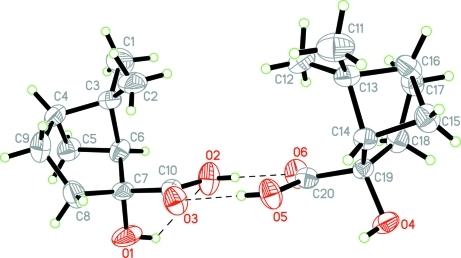
The asymmetric unit of the title compound. Displacement ellipsoids are drawn at the 30% probability level. Hydrogen bonds are shown as dashed lines.

**Fig. 2.**
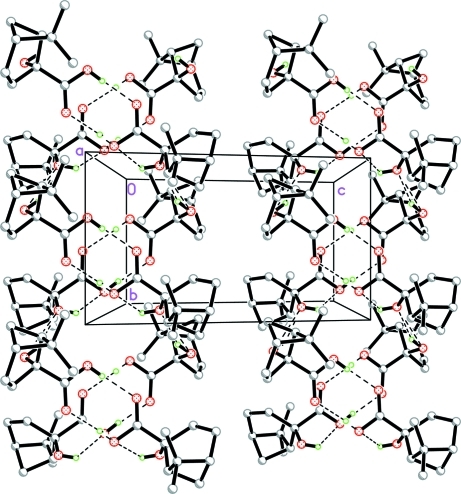
Crystal packing of the title compound, viewed along the a axis. Hydrogen bonds are shown as dashed lines.

## supplementary crystallographic information

### Comment

Terpenes are convenient chiral precursors due to their availability and low cost, and among them β-pinene is an important material. Many valuable chemicals were prepared from β-pinene. For instance, nopinone (Winstein & Holness, 1955) and nopinic acid were prepared by oxidation of β-pinene. Although the title compound has been prepared (Ma *et al.*,  2007) and the crystal structure of sodium nopinate has been reported (Ma *et al.*, 2008), the crystal structure of nopinic acid has not been reported. In this paper, we report the crystal structure of the title compound.

The asymmetric unit contains two crystallographically independent molecules (Fig. 1) with similar geometry. The six-membered rings in both the molecules adopt envelope conformations. The independent molecules are linked through a pair of O–H···O hydrogen bonds (Table 1) forming a dimer. The dimers are linked into helical chains along the *b* axis (Fig. 2) by O—H···O hydrogen bonds.

### Experimental

Potassium permanganate (12.0 g) and NaOH (1.5 g) were dissolved in the mixture of water (100 ml) and t-butylalcohol (50 ml). While stirring vigorously, pure (-)-beta-pinene (5.2 g) was added. The reaction was maintained at the temperature of 288–298 K for 0.5 h. The mixture was heated to 353 K, then filtered and the precipitate was washed with hot water. After standing for 12 h at 273 K, sodium nopinate was filtered. The crude sodium nopinate was acidified with dilute hydrochloric acid and extracted with dichloromethane, then the product, crude nopinic acid was obtained. The crude nopinic acid was recrystallized from toluene. Single crystals of the title compound suitable for X-ray diffraction were obtained by slow evaporation of a ethanol-toluene solution.

### Refinement

H atoms of hydroxyl groups were located in a difference map and their parameters were refined with a O-H distance restraint of 0.82 (1) Å. The remaining H atoms were positioned geometrically [O-H = 0.82 Å and C-H = 0.96–0.98 Å] and included in the refinement in the riding motion approximation, with *U*_iso_(H) = 1.2 or 1.5*U*_eq_ of the carrier atom. In the absence of significant anomalous scattering, Friedel pairs were merged prior to the final refinement.

### Crystal data

**Table:**

C_10_H_16_O_3_	*F*(000) = 800
*M**_r_* = 184.23	*D*_x_ = 1.210 Mg m^−^^3^
Monoclinic, *C*2	Mo *K*α radiation, λ = 0.71073 Å
Hall symbol: C 2y	Cell parameters from 25 reflections
*a* = 26.796 (5) Å	θ = 10–13°
*b* = 6.6560 (13) Å	µ = 0.09 mm^−^^1^
*c* = 12.250 (3) Å	*T* = 293 K
β = 112.23 (3)°	Block, colourless
*V* = 2022.5 (9) Å^3^	0.30 × 0.20 × 0.20 mm
*Z* = 8	

### Data collection

**Table:**

Enraf–Nonius CAD-4 diffractometer	1565 reflections with *I* > 2σ(*I*)
Radiation source: fine-focus sealed tube	*R*_int_ = 0.018
graphite	θ_max_ = 25.3°, θ_min_ = 1.6°
ω/2θ scans	*h* = 0→32
Absorption correction: ψ scan (*XCAD4*; Harms & Wocadlo, 1995)	*k* = 0→7
*T*_min_ = 0.974, *T*_max_ = 0.983	*l* = −14→13
2047 measured reflections	3 standard reflections every 200 reflections
2002 independent reflections	intensity decay: 1%

### Refinement

**Table:**

Refinement on *F*^2^	Secondary atom site location: difference Fourier map
Least-squares matrix: full	Hydrogen site location: inferred from neighbouring sites
*R*[*F*^2^ > 2σ(*F*^2^)] = 0.056	H atoms treated by a mixture of independent and constrained refinement
*wR*(*F*^2^) = 0.153	*w* = 1/[σ^2^(*F*_o_^2^) + (0.1*P*)^2^ + 0.3*P*] where *P* = (*F*_o_^2^ + 2*F*_c_^2^)/3
*S* = 1.00	(Δ/σ)_max_ = 0.001
2002 reflections	Δρ_max_ = 0.21 e Å^−^^3^
242 parameters	Δρ_min_ = −0.23 e Å^−^^3^
3 restraints	Extinction correction: *SHELXL97* (Sheldrick, 2008), Fc^*^=kFc[1+0.001xFc^2^λ^3^/sin(2θ)]^-1/4^
Primary atom site location: structure-invariant direct methods	Extinction coefficient: 0.044 (4)

### Special details

**Table:**

Geometry. All e.s.d.'s (except the e.s.d. in the dihedral angle between two l.s. planes) are estimated using the full covariance matrix. The cell e.s.d.'s are taken into account individually in the estimation of e.s.d.'s in distances, angles and torsion angles; correlations between e.s.d.'s in cell parameters are only used when they are defined by crystal symmetry. An approximate (isotropic) treatment of cell e.s.d.'s is used for estimating e.s.d.'s involving l.s. planes.
Refinement. Refinement of *F*^2^ against ALL reflections. The weighted *R*-factor *wR* and goodness of fit *S* are based on *F*^2^, conventional *R*-factors *R* are based on *F*, with *F* set to zero for negative *F*^2^. The threshold expression of *F*^2^ > σ(*F*^2^) is used only for calculating *R*-factors(gt) *etc*. and is not relevant to the choice of reflections for refinement. *R*-factors based on *F*^2^ are statistically about twice as large as those based on *F*, and *R*- factors based on ALL data will be even larger.

### Fractional atomic coordinates and isotropic or equivalent isotropic displacement parameters (Å^2^)

**Table:**

	*x*	*y*	*z*	*U*_iso_*/*U*_eq_	
O1	0.03238 (12)	0.4845 (8)	0.2047 (3)	0.0997 (14)	
H1A	0.021 (3)	0.504 (15)	0.134 (2)	0.150*	
C1	0.2225 (3)	0.6237 (14)	0.2710 (5)	0.116 (2)	
H1B	0.2158	0.7519	0.2986	0.174*	
H1C	0.2171	0.6336	0.1891	0.174*	
H1D	0.2590	0.5836	0.3156	0.174*	
O2	0.07227 (15)	0.3644 (5)	0.0307 (3)	0.0842 (10)	
H2D	0.0677	0.2896	−0.0255	0.126*	
C2	0.1946 (2)	0.2647 (10)	0.2414 (5)	0.0941 (19)	
H2A	0.1946	0.2814	0.1635	0.141*	
H2B	0.1672	0.1701	0.2391	0.141*	
H2C	0.2292	0.2157	0.2931	0.141*	
O3	0.06567 (14)	0.0776 (5)	0.1168 (3)	0.0724 (9)	
C3	0.18336 (16)	0.4660 (9)	0.2865 (4)	0.0672 (12)	
C4	0.18223 (17)	0.4726 (8)	0.4114 (4)	0.0681 (12)	
H4A	0.2164	0.5083	0.4747	0.082*	
C5	0.14121 (19)	0.6413 (8)	0.3696 (4)	0.0732 (13)	
H5A	0.1569	0.7746	0.3781	0.088*	
H5B	0.1130	0.6356	0.4009	0.088*	
C6	0.12546 (16)	0.5514 (7)	0.2461 (3)	0.0601 (10)	
H6A	0.1162	0.6498	0.1820	0.072*	
C7	0.08375 (14)	0.3881 (7)	0.2299 (3)	0.0571 (10)	
C8	0.0964 (2)	0.2662 (9)	0.3430 (4)	0.0835 (16)	
H8A	0.0901	0.1252	0.3223	0.100*	
H8B	0.0714	0.3056	0.3794	0.100*	
C9	0.1543 (2)	0.2906 (9)	0.4343 (4)	0.0841 (16)	
H9A	0.1533	0.3013	0.5124	0.101*	
H9B	0.1750	0.1716	0.4330	0.101*	
C10	0.07381 (16)	0.2592 (7)	0.1207 (4)	0.0542 (10)	
O4	−0.00459 (10)	−0.2034 (5)	−0.3282 (2)	0.0640 (8)	
H4C	−0.010 (2)	−0.270 (8)	−0.276 (4)	0.096*	
O5	0.05228 (13)	−0.1491 (5)	−0.0754 (2)	0.0677 (8)	
H5C	0.0566	−0.0769	−0.0183	0.102*	
O6	0.05719 (14)	0.1444 (5)	−0.1566 (3)	0.0714 (9)	
C11	0.1765 (2)	−0.5132 (10)	−0.1362 (6)	0.0972 (18)	
H11A	0.1808	−0.5215	−0.0549	0.146*	
H11B	0.2114	−0.5142	−0.1416	0.146*	
H11C	0.1559	−0.6262	−0.1785	0.146*	
C12	0.17137 (18)	−0.1448 (10)	−0.1071 (4)	0.0769 (14)	
H12A	0.1733	−0.1796	−0.0295	0.115*	
H12B	0.1495	−0.0269	−0.1339	0.115*	
H12C	0.2070	−0.1186	−0.1048	0.115*	
C13	0.14673 (15)	−0.3165 (7)	−0.1904 (4)	0.0591 (11)	
C14	0.08439 (14)	−0.3538 (6)	−0.2409 (3)	0.0480 (9)	
H14A	0.0720	−0.4534	−0.1978	0.058*	
C15	0.08937 (19)	−0.4313 (7)	−0.3542 (4)	0.0660 (11)	
H15A	0.0597	−0.3933	−0.4260	0.079*	
H15B	0.0973	−0.5737	−0.3532	0.079*	
C16	0.13896 (18)	−0.2949 (7)	−0.3219 (4)	0.0650 (12)	
H16A	0.1685	−0.3484	−0.3420	0.078*	
C17	0.1211 (2)	−0.0838 (8)	−0.3660 (4)	0.0675 (13)	
H17A	0.1174	−0.0727	−0.4476	0.081*	
H17B	0.1483	0.0115	−0.3200	0.081*	
C18	0.06712 (17)	−0.0321 (7)	−0.3564 (3)	0.0599 (11)	
H18A	0.0681	0.1080	−0.3339	0.072*	
H18B	0.0388	−0.0464	−0.4339	0.072*	
C19	0.05197 (13)	−0.1576 (6)	−0.2696 (3)	0.0461 (9)	
C20	0.05493 (15)	−0.0405 (6)	−0.1616 (3)	0.0509 (9)	

### Atomic displacement parameters (Å^2^)

**Table:**

	*U*^11^	*U*^22^	*U*^33^	*U*^12^	*U*^13^	*U*^23^
O1	0.0457 (16)	0.123 (3)	0.119 (3)	0.014 (2)	0.0179 (17)	−0.057 (3)
C1	0.100 (4)	0.146 (7)	0.110 (4)	−0.050 (5)	0.050 (3)	−0.019 (5)
O2	0.131 (3)	0.0553 (19)	0.0582 (17)	−0.002 (2)	0.0263 (16)	−0.0013 (17)
C2	0.056 (3)	0.120 (5)	0.104 (4)	0.024 (3)	0.028 (3)	−0.017 (4)
O3	0.099 (2)	0.0558 (19)	0.0706 (19)	−0.0094 (18)	0.0413 (17)	−0.0104 (16)
C3	0.046 (2)	0.084 (3)	0.071 (3)	0.000 (2)	0.0214 (18)	−0.005 (3)
C4	0.052 (2)	0.080 (3)	0.058 (2)	−0.005 (2)	0.0052 (17)	−0.004 (2)
C5	0.071 (3)	0.066 (3)	0.076 (3)	−0.005 (3)	0.020 (2)	−0.023 (3)
C6	0.063 (2)	0.049 (2)	0.057 (2)	0.010 (2)	0.0102 (17)	0.001 (2)
C7	0.042 (2)	0.065 (3)	0.064 (2)	0.008 (2)	0.0191 (16)	−0.013 (2)
C8	0.101 (4)	0.093 (4)	0.067 (3)	−0.035 (3)	0.044 (3)	−0.019 (3)
C9	0.108 (4)	0.085 (4)	0.051 (2)	−0.004 (3)	0.021 (2)	0.007 (3)
C10	0.051 (2)	0.055 (3)	0.055 (2)	0.0081 (19)	0.0183 (18)	−0.0031 (19)
O4	0.0449 (14)	0.072 (2)	0.0648 (16)	−0.0111 (15)	0.0095 (12)	0.0054 (16)
O5	0.103 (2)	0.0507 (17)	0.0607 (16)	−0.0033 (17)	0.0441 (15)	0.0021 (15)
O6	0.103 (2)	0.0429 (18)	0.0730 (19)	−0.0012 (17)	0.0385 (17)	−0.0001 (15)
C11	0.076 (3)	0.086 (4)	0.126 (5)	0.022 (3)	0.034 (3)	0.021 (4)
C12	0.053 (3)	0.090 (4)	0.072 (3)	−0.004 (3)	0.006 (2)	−0.001 (3)
C13	0.045 (2)	0.059 (3)	0.071 (2)	0.003 (2)	0.0190 (17)	0.000 (2)
C14	0.051 (2)	0.0404 (19)	0.055 (2)	−0.0065 (18)	0.0232 (16)	0.0045 (18)
C15	0.083 (3)	0.049 (2)	0.073 (3)	−0.011 (2)	0.037 (2)	−0.014 (2)
C16	0.070 (3)	0.062 (3)	0.078 (3)	−0.006 (2)	0.046 (2)	−0.007 (2)
C17	0.085 (3)	0.065 (3)	0.062 (2)	−0.022 (2)	0.038 (2)	−0.003 (2)
C18	0.069 (3)	0.054 (2)	0.054 (2)	−0.008 (2)	0.0204 (18)	0.007 (2)
C19	0.0426 (18)	0.047 (2)	0.0460 (18)	−0.0062 (17)	0.0140 (14)	0.0038 (17)
C20	0.049 (2)	0.046 (3)	0.059 (2)	0.0046 (18)	0.0217 (17)	0.0058 (19)

### Geometric parameters (Å, °)

**Table:**

O1—C7	1.442 (5)	O4—C19	1.443 (4)
O1—H1A	0.82 (2)	O4—H4C	0.84 (5)
C1—C3	1.546 (8)	O5—C20	1.303 (5)
C1—H1B	0.96	O5—H5C	0.82
C1—H1C	0.96	O6—C20	1.232 (5)
C1—H1D	0.96	C11—C13	1.547 (8)
O2—C10	1.294 (5)	C11—H11A	0.96
O2—H2D	0.82	C11—H11B	0.96
C2—C3	1.523 (8)	C11—H11C	0.96
C2—H2A	0.96	C12—C13	1.508 (7)
C2—H2B	0.96	C12—H12A	0.96
C2—H2C	0.96	C12—H12B	0.96
O3—C10	1.226 (6)	C12—H12C	0.96
C3—C4	1.542 (6)	C13—C16	1.550 (6)
C3—C6	1.547 (6)	C13—C14	1.566 (5)
C4—C9	1.505 (8)	C14—C15	1.533 (5)
C4—C5	1.518 (7)	C14—C19	1.534 (5)
C4—H4A	0.98	C14—H14A	0.98
C5—C6	1.530 (6)	C15—C16	1.533 (6)
C5—H5A	0.97	C15—H15A	0.97
C5—H5B	0.97	C15—H15B	0.97
C6—C7	1.517 (6)	C16—C17	1.517 (7)
C6—H6A	0.98	C16—H16A	0.98
C7—C10	1.526 (6)	C17—C18	1.533 (6)
C7—C8	1.529 (7)	C17—H17A	0.97
C8—C9	1.540 (7)	C17—H17B	0.97
C8—H8A	0.97	C18—C19	1.523 (5)
C8—H8B	0.97	C18—H18A	0.97
C9—H9A	0.97	C18—H18B	0.97
C9—H9B	0.97	C19—C20	1.512 (5)
			
C7—O1—H1A	103 (6)	C19—O4—H4C	100 (4)
C3—C1—H1B	109.5	C20—O5—H5C	109.5
C3—C1—H1C	109.5	C13—C11—H11A	109.5
H1B—C1—H1C	109.5	C13—C11—H11B	109.5
C3—C1—H1D	109.5	H11A—C11—H11B	109.5
H1B—C1—H1D	109.5	C13—C11—H11C	109.5
H1C—C1—H1D	109.5	H11A—C11—H11C	109.5
C10—O2—H2D	109.5	H11B—C11—H11C	109.5
C3—C2—H2A	109.5	C13—C12—H12A	109.5
C3—C2—H2B	109.5	C13—C12—H12B	109.5
H2A—C2—H2B	109.5	H12A—C12—H12B	109.5
C3—C2—H2C	109.5	C13—C12—H12C	109.5
H2A—C2—H2C	109.5	H12A—C12—H12C	109.5
H2B—C2—H2C	109.5	H12B—C12—H12C	109.5
C2—C3—C4	117.8 (5)	C12—C13—C11	109.2 (4)
C2—C3—C1	108.4 (4)	C12—C13—C16	119.0 (4)
C4—C3—C1	111.8 (4)	C11—C13—C16	111.6 (4)
C2—C3—C6	121.3 (4)	C12—C13—C14	121.0 (4)
C4—C3—C6	85.0 (3)	C11—C13—C14	109.6 (4)
C1—C3—C6	110.9 (5)	C16—C13—C14	84.3 (3)
C9—C4—C5	108.1 (4)	C15—C14—C19	108.3 (3)
C9—C4—C3	111.1 (4)	C15—C14—C13	88.0 (3)
C5—C4—C3	88.3 (3)	C19—C14—C13	112.5 (3)
C9—C4—H4A	115.4	C15—C14—H14A	115.0
C5—C4—H4A	115.4	C19—C14—H14A	115.0
C3—C4—H4A	115.4	C13—C14—H14A	115.0
C4—C5—C6	86.4 (3)	C14—C15—C16	86.0 (3)
C4—C5—H5A	114.2	C14—C15—H15A	114.3
C6—C5—H5A	114.2	C16—C15—H15A	114.3
C4—C5—H5B	114.2	C14—C15—H15B	114.3
C6—C5—H5B	114.2	C16—C15—H15B	114.3
H5A—C5—H5B	111.4	H15A—C15—H15B	111.5
C7—C6—C5	108.9 (4)	C17—C16—C15	109.3 (4)
C7—C6—C3	112.2 (4)	C17—C16—C13	110.8 (4)
C5—C6—C3	87.7 (3)	C15—C16—C13	88.6 (3)
C7—C6—H6A	115.0	C17—C16—H16A	115.1
C5—C6—H6A	115.0	C15—C16—H16A	115.1
C3—C6—H6A	115.0	C13—C16—H16A	115.1
O1—C7—C6	107.8 (4)	C16—C17—C18	111.1 (4)
O1—C7—C10	103.1 (3)	C16—C17—H17A	109.4
C6—C7—C10	113.1 (3)	C18—C17—H17A	109.4
O1—C7—C8	107.2 (4)	C16—C17—H17B	109.4
C6—C7—C8	111.2 (3)	C18—C17—H17B	109.4
C10—C7—C8	113.7 (4)	H17A—C17—H17B	108.0
C7—C8—C9	114.7 (4)	C19—C18—C17	116.0 (4)
C7—C8—H8A	108.6	C19—C18—H18A	108.3
C9—C8—H8A	108.6	C17—C18—H18A	108.3
C7—C8—H8B	108.6	C19—C18—H18B	108.3
C9—C8—H8B	108.6	C17—C18—H18B	108.3
H8A—C8—H8B	107.6	H18A—C18—H18B	107.4
C4—C9—C8	112.7 (4)	O4—C19—C20	104.0 (3)
C4—C9—H9A	109.1	O4—C19—C18	106.0 (3)
C8—C9—H9A	109.1	C20—C19—C18	112.9 (3)
C4—C9—H9B	109.1	O4—C19—C14	109.3 (3)
C8—C9—H9B	109.1	C20—C19—C14	113.6 (3)
H9A—C9—H9B	107.8	C18—C19—C14	110.5 (3)
O3—C10—O2	123.6 (4)	O6—C20—O5	122.0 (4)
O3—C10—C7	123.9 (4)	O6—C20—C19	122.9 (4)
O2—C10—C7	112.4 (4)	O5—C20—C19	115.0 (3)
			
C2—C3—C4—C9	−40.5 (5)	C12—C13—C14—C15	147.2 (4)
C1—C3—C4—C9	−167.1 (5)	C11—C13—C14—C15	−84.4 (4)
C6—C3—C4—C9	82.4 (4)	C16—C13—C14—C15	26.6 (3)
C2—C3—C4—C5	−149.2 (4)	C12—C13—C14—C19	38.2 (5)
C1—C3—C4—C5	84.2 (5)	C11—C13—C14—C19	166.6 (4)
C6—C3—C4—C5	−26.3 (3)	C16—C13—C14—C19	−82.4 (3)
C9—C4—C5—C6	−85.0 (4)	C19—C14—C15—C16	86.2 (3)
C3—C4—C5—C6	26.6 (3)	C13—C14—C15—C16	−26.9 (3)
C4—C5—C6—C7	86.2 (4)	C14—C15—C16—C17	−84.5 (4)
C4—C5—C6—C3	−26.5 (4)	C14—C15—C16—C13	27.2 (3)
C2—C3—C6—C7	36.3 (5)	C12—C13—C16—C17	−39.0 (5)
C4—C3—C6—C7	−83.3 (4)	C11—C13—C16—C17	−167.6 (4)
C1—C3—C6—C7	165.2 (4)	C14—C13—C16—C17	83.5 (4)
C2—C3—C6—C5	145.8 (5)	C12—C13—C16—C15	−149.1 (4)
C4—C3—C6—C5	26.1 (4)	C11—C13—C16—C15	82.2 (4)
C1—C3—C6—C5	−85.4 (4)	C14—C13—C16—C15	−26.6 (3)
C5—C6—C7—O1	78.6 (4)	C15—C16—C17—C18	35.4 (5)
C3—C6—C7—O1	174.0 (3)	C13—C16—C17—C18	−60.7 (4)
C5—C6—C7—C10	−168.0 (4)	C16—C17—C18—C19	20.9 (5)
C3—C6—C7—C10	−72.7 (4)	C17—C18—C19—O4	−137.5 (4)
C5—C6—C7—C8	−38.6 (5)	C17—C18—C19—C20	109.3 (4)
C3—C6—C7—C8	56.7 (5)	C17—C18—C19—C14	−19.2 (5)
O1—C7—C8—C9	−134.0 (5)	C15—C14—C19—O4	77.7 (3)
C6—C7—C8—C9	−16.4 (6)	C13—C14—C19—O4	173.3 (3)
C10—C7—C8—C9	112.7 (5)	C15—C14—C19—C20	−166.6 (3)
C5—C4—C9—C8	38.3 (6)	C13—C14—C19—C20	−71.0 (4)
C3—C4—C9—C8	−57.0 (6)	C15—C14—C19—C18	−38.6 (4)
C7—C8—C9—C4	16.9 (7)	C13—C14—C19—C18	57.0 (4)
O1—C7—C10—O3	−101.6 (5)	O4—C19—C20—O6	−99.0 (5)
C6—C7—C10—O3	142.3 (5)	C18—C19—C20—O6	15.5 (5)
C8—C7—C10—O3	14.1 (6)	C14—C19—C20—O6	142.2 (4)
O1—C7—C10—O2	75.2 (5)	O4—C19—C20—O5	77.9 (4)
C6—C7—C10—O2	−40.9 (5)	C18—C19—C20—O5	−167.7 (3)
C8—C7—C10—O2	−169.1 (4)	C14—C19—C20—O5	−40.9 (4)

### Hydrogen-bond geometry (Å, °)

**Table:**

*D*—H···*A*	*D*—H	H···*A*	*D*···*A*	*D*—H···*A*
O1—H1A···O2	0.81 (3)	2.38 (8)	2.837 (5)	116 (7)
O2—H2D···O6	0.82	1.80	2.621 (5)	175
O4—H4C···O1^i^	0.84 (5)	2.05 (5)	2.830 (5)	156 (5)
O5—H5C···O3	0.82	1.88	2.704 (4)	177

Symmetry codes: (i) −*x*, *y*−1, −*z*.
